# Serum very long-chain fatty acids (VLCFA) levels as predictive biomarkers of diseases severity and probability of survival in peroxisomal disorders

**DOI:** 10.1371/journal.pone.0238796

**Published:** 2020-09-18

**Authors:** Teresa Joanna Stradomska, Małgorzata Syczewska, Ewa Jamroz, Agata Pleskaczyńska, Piotr Kruczek, Elżbieta Ciara, Anna Tylki-Szymanska

**Affiliations:** 1 Department of Biochemistry, Radioimmunology & Experimental Medicine, Children’s Memorial Health Institute, Warsaw, Poland; 2 Department of Pediatrics Rehabilitation, Children’s Memorial Health Institute, Warsaw, Poland; 3 Department of Pediatric and Neurology of Developmental Age, Clinical Hospital No. 6 of Medical University of Silesia, Katowice, Poland; 4 Department of Neonatology, Pathology and Neonatal Intensive Care Unit, Children’s Memorial Health Institute, Warsaw, Poland; 5 Department of Neonatal Pathology and Intensive Care Unit, University Children’s Hospital of Cracow, Cracow, Poland; 6 Department of Medical Genetics, Children’s Memorial Health Institute, Warsaw, Poland; 7 Department of Pediatrics, Nutrition and Metabolic Disease, Children’s Memorial Health Institute, Warsaw, Poland; Imagine Institute, FRANCE

## Abstract

Peroxisomal disorders (PD) are a heterogeneous group of rare diseases caused by a defect in peroxisome biogenesis or a disruption of a peroxisomal function at a single enzyme or at transporter level. The main biochemical markers for PD are very long-chain fatty acids (VLCFA). The aim of the study was to investigate the correlation of basic diagnostic parameter, i.e. VLCFA, with disease severity, determined through the survival time. We performed a retrospective study in patients with PD (n = 31; aged 1 week—21 years). Evaluation of VLCFA results from patients were as follows: 15 patients with classical Zellweger syndrome (ZS), 3 patients with mild outcome of ZS, 9 individuals with D-Bifunctional Protein Deficiency (DBP), and no specified results in the case of 4 patients. Patients with classical ZS had higher VLCFA levels, compared to individuals with mild form of ZS and also to patients with DBP; for **C26:0/C22:0:** 0.65±0.18; 0.11±0.09; 0.30±0.13 *(P* < 0.001) and for **C26:0:** 5.20±1.78; 0.76±0.46; 2.61±0.97[mg/mL] (*P* < 0.001) respectively. The only variable parameter, i.e. the one that determines the survival time of patients, was **C26:0** (Chi^2^ = 19,311, *P* < 0.0001). Correlation coefficient between survival time and **C26:0** level was statistically significant (*r* = -0.762), and the results showed that high levels of **C26:0** were associated with short survival time.

**Conclusion:**

VLCFA levels correlate with the severity of the clinical course of ZS, DBP and mild ZSD. The best predictive value for estimating the projected disease severity and survival time is a concentration of C26:0.

## Introduction

Peroxisomes, single-membrane organelles, are a part of the structure of all eukaryotes, except for the red blood cells. Over 50 proteins participate in numerous anabolic and catabolic functions of the peroxisomal apparatus. Peroxisomes are the site of the synthesis of cholesterol, bile acids, polyunsaturated fat acids (FA), as well as of plasmalogen. The main peroxisomal degradation processes are α- and β –oxidation (phytanic acid, very long-chain fatty acid (VLCFA) and pristanic acid). Peroxisomes play fundamental role in redox cellular homeostasis by antioxidant enzymes inactivating reactive oxygen species (ROS) [1–4]. So far, 14 *PEX* genes responsible for the structure of peroxisomal proteins have been identified in human. Peroxisomal disorders (PD) are a large group of inborn errors of metabolism, being a consequence of the defect in at least one of the *PEX* genes–peroxisome biogenesis disorders (PBD) or non- *PEX genes*–which result in a range of disorders with single enzyme deficiency (SED) [2, 5]. PBD are autosomal recessive disorders with an estimated incidence of 1:50.000 to 1:500.000 newborns [6–8]. Currently, this group includes two clinically distinct phenotypes: the Zellweger spectrum disorders (ZSDs; OMIM #601539) and Rhizomelic chondrodysplasia punctata t. I (RCDP; OMIM #215100). The majority of ZSDs patients presented a well-known classical phenotype, namely Zellweger syndrome (ZS), with severe and early symptoms (congenital malformations, liver dysfunction, severe hypotonic, ocular anomalies [4, 9]. Nevertheless, cases with later onset in childhood, and even with mild clinical form in adulthood, are more frequently reported [10, 11]. The group of single enzyme deficiency results in the loss of a single peroxisomal function and includes more than 10 disorders, out of which the most common ones are: X-linked adrenoleukodystrophy (X-ALD; OMIM #300100) and D-bifunctional protein deficiency (DBP; OMIM #261515), with the frequency of 1:17.000 and 1:100.000 respectively [12, 13]. The defect of peroxisomal functions consequently leads to changes in the levels of relevant metabolites detected in body fluids. Their detection is the basis for diagnostics at the biochemical level. VLCFA was the first biochemical marker identified for peroxisomal diseases. VLCFA (> C22) are exclusively degraded in the peroxisomal β-oxidation process. A defect in the process at one of the stages leads to the accumulation of this biochemical marker in body fluids and tissues [14]. In this study, we examine the correlation of VLCFA levels with disease severity defined as survival. Patients with X-ALD were excluded from this analysis because no statistically significant differences in VLCFA levels between severe childhood cerebral X-ALD and milder AMN, with several times longer survival, have been shown [8], which suggests the influence of other factors on the phenotype of the disease and thus life expectancy.

## Materials and methods

### Subjects

The study includes retrospective data from patients who were referred to our institute for suspicion of peroxisomal disorders (PDs) between 1994 and 2017. The patients demonstrated a wide range of disorders that involved many organs; craniofacial dysmorphia, hypotonic, seizures, psychomotor delay, impaired hearing and seeing, hepatomegaly, malformations in CNS. The diagnosis of PD was based on the high concentration of VLCFA in serum or by DNA analysis. Age at diagnosis was defined as age biochemically confirmed diagnosis. All the patients came from non-consanguineous parents. VLCFA results of 31 patients, from 28 families (12 women and 19 men) with peroxisomal disorders were investigated: 15 with classical ZS, 3 with mild outcome of ZS and 9 with DBP, 4 –unspecified.

### VLCFA analysis

VLCFA levels were analysed on serum samples by GC method as described before [8]. A total of 119 biochemical analyses were performed, ranking from 2 to 5 tests per patient. The results are expressed as mean ± SD.

### Statistical analysis

The data analysis was performed using survival analysis method: Cox proportional-hazards method was used to identify variables predicting the patients’ survival time. All patients were included in the statistical model. The following variables were taken into account: age at the diagnosis, C22:0, C24:0, C26:0, C24:0/C22:0, C26:0/C22:0 and type of the syndrome. Additionally, the Spearman rank correlation test was used as the data was non-normally distributed (Kolmogorov-Smirnow test was used to check the type of the distribution). The cut-off level for *P*—value was 0.05. All calculations were done in STATISTICA 10.0.

The study is retrospective and only anonymised data was used. Written informed consent was obtained from one patient and parents of all other patients.

## Results

The clinical manifestation was present soon after birth in most patients (except one mild form of ZSD). The age of biochemical diagnosis was between 10 days and 20 years. The biochemical diagnosis was carried out in the course from 10 days to 6 months for ZS; 1–8 months for DBP and 3–20 years for ZSD mild. The mean age diagnosis was (mean ±SD) 2.2 ±2.2 months. Serum VLCFA levels as C22:0, C24:0, C26:0, C24:0/C22:0, C26:0/C22:0 were measurements used according to the earlier described method. Accumulation of serum VLCFA (mean ±SD) in patients with classical severe ZS was higher than in patients with mild form of ZS and in patients with DBP, for C26:0/C22:0 0.65±0.18; 0.11±0.09; 0.30±013 (*P* < 0.001) and for C26:0 [mg/mL] 5.20±1.78; 07.4±0.46; 2.61±0.97 (*P* < 0.001) respectively (Table 1). The range of survival for patients from particular groups varied widely. It was very low in group I with classical ZS (2–12 months), higher in DBP patients (1–3y), and the highest for mild form ZSDs (3-20y).

**Table 1:** VLCFA levels, age diagnosis and survival in patients with peroxisomal disorders.

*Patients*	*Age of diagnosis (range*, *mo*.)	*C26*:*0 [mg/mL]*	*C24*:*0/C22*:*0*	*C26*:*0/C22*:*0*	*Survival (range*, *mo*.*)*
ZS (15)	2.0 ±2.1 (0,2–6)	5.20 ± 1.78 (3.32–9.93)	2.23 ± 0.31 (1.72–2.95)	0.65± 0.18 (0.40–1.03)	6.1 ± 2.9 (2–12)
Mild ZS (3)	101 ± 120 (28–240)	0.76 ± 0.46 (0.32–1.24)	1.22 ± 0.27 (0.96–1.50)	0.11± 0.09 (0.025–0.211)	125 ± 132,5 (28–276)
DBP (9)	2.8 ±2.5 (0,5–8)	2.61 ± 0.97 (1.56–3.45)	2.02 ± 0.23 (1.60–2.38)	0.30 ± 0.13 (0.14–0.47)	22.3 ± 7.5 (12–36)
unspecified PD (4)	1.4 ± 1.7 (0,5–4)	3.64 ± 0.64 (3.15–4.59)	2.01 ± 0.49 (1.57–2.49)	0.44 ± 0.03 (0.40–0.48)	11.1 ± 5.0 (6–18)
Control	(—)	0.15 ± 0.05	0.782 ± 0.06	0.008 ± 0.05	(—)

Data is mean ± SD.

**Fig 1:**
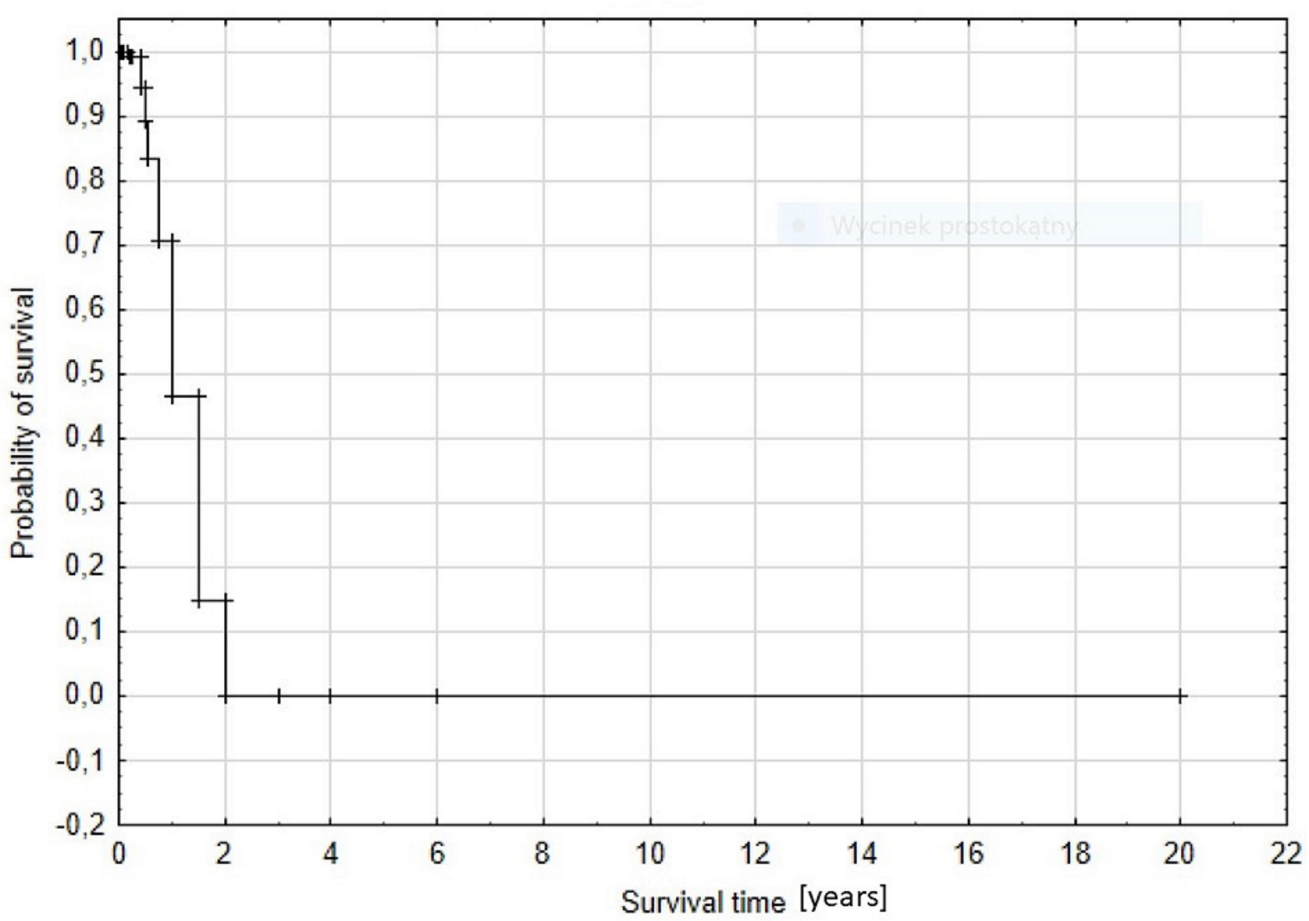
Survival analysis from Cox proportional model, showing the probability of survival depending on time.

**Fig 2:**
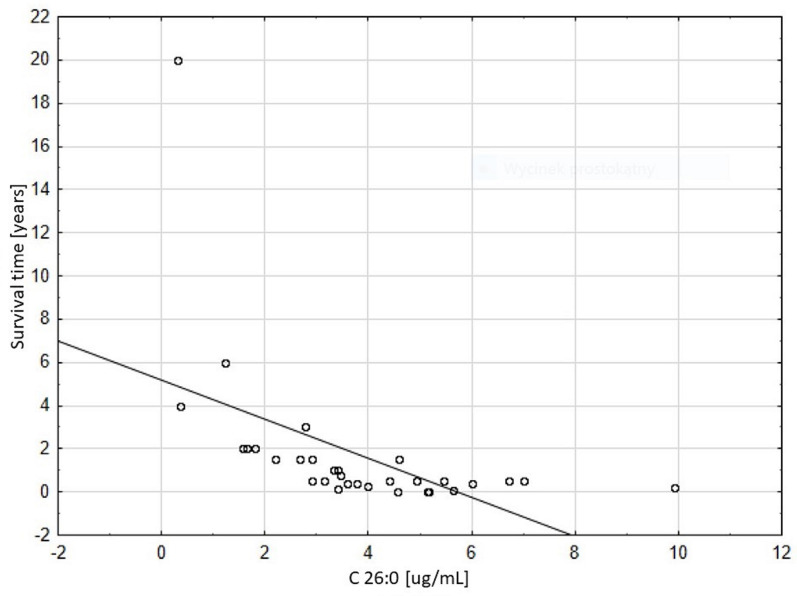
Dependence of the survival time on C26:0 level (Spearman rank correlation is statistically significant, R = -0.762).

The Cox method revealed that C26:0 (chi^2^ = 19,311, *P* < 0.0001) was the only variable affecting time of the patients’ survival. The relative risk (hazard ratio) calculated from the model was 2.10, while 95% confidence limits were: lower: 1.46, and upper 3.02. The Spearman rank correlation coefficient between survival time and C26:0 level was statistically significant and strong (*r* = -0.762), and it showed that high levels of C26:0 were associated with shorter survival time (Figs 1 and 2). Table 2 presents clinical manifestation and VLCFA levels in patients with mild ZSDs phenotype. Although dysmorphic features, delayed psychomotor development, hearing and visual impairment were observed in all patients, these symptoms appeared at different ages and severities. Patients P.1 and P.3 presented these symptoms in the most severe and lightest degree, respectively. For example, hearing loss has been present since birth (P.1), occurred about 1 year of age (P.2) and at the age of 4 years (P.3). A mutation with a milder phenotype described earlier has been identified in Patient 2 [15]. In turn, mutations found in P.1 and P. 3 are the ones that are newly described, and due to the presented clinical picture and length of life (P.1 –died at 6 y), (P.3—currently 23 y) should be thought to cause PD with milder course than classical ZS [11, 16]. The data presented indicates that milder clinical symptoms correspond to lower VLCFA levels and longer survival.

**Table 2:** Clinical manifestation and VLCFA levels in patients with mild clinical form of ZSD.

Patient	Psychomotor development	Dysmorphic features	Hearing impairment	Visual impairment	Speech	Severity of clinical manife-station	Mutations	C26:0/C22:0 N<0.02	C26:0 N<0.25	Value of VLCFA levels	Survival (years)
P.1	Delayed 20 mo. stood in bed 2.5y regress no walk	high forehead, broad nasal bridge, hypoplastic supraorbital ridges microcephaly	since birth + 8 mo. aid hearing	hypoplastic optic disks, nystagmus, strabismus.	1y babbling Absent	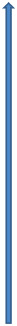	*PEX 6* p.Ala94 Pro	0.211	1.24	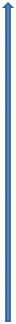	3 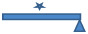 6 died
P.2	Delayed ^9^/_12_ y diminish ataxia	high forehead, broad nasal bridge, low set eyes, ears	1 y +	strabismus. nystagmus	Incomprehen-sible sounds		*PEX 1* c.2528G>A p.Gly843Asp)	0.101	0.730		2^4^/_12_ 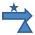 2^6^/_12_ alive
P.3	Delayed 3.5 y walk alone 12 y progressive spastic paresis 21 y wheelchair	Only microcephaly	4 y + 5 y aid hearing	1.5 y nystagmus 18 y bilateral cataracts			*PEX 1* c.1769T>C, p.Leu590Pro; c.3450T>A, p.Cys1150	0.025	0.315		21 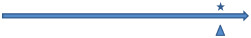 23 y alive

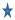
*N*–references values

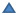
 Age of diagnosis, Survival (years)

**Fig 3:**
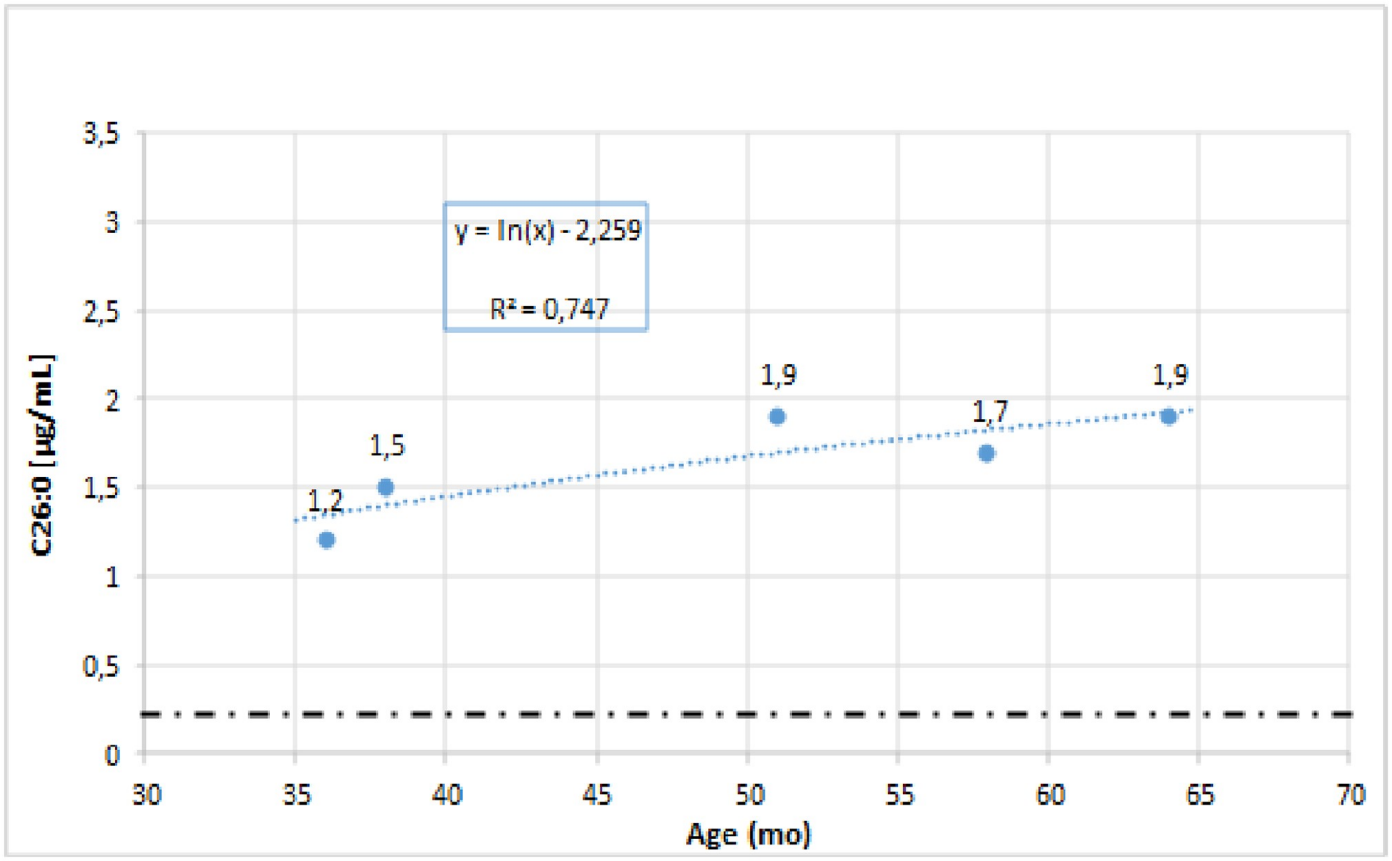
Long-time monitoring of serum C26:0 levels in patient with ZSD (P.1).

VLCFA levels were monitored in 4 patients (2 with ZS, 1 with DBP and ZSD) [11, 17]. Long- time monitoring of the concentration of C26:0 in serum is presented in Fig 3.

## Discussion

Numerous metabolic processes taking place in peroxisomes, underline their important role in cell metabolism. The fact that peroxisomes are of key importance for human health and development is evidenced by a group of genetic diseases in which peroxisomes are partially or even completely damaged. Defect in the structure of these microorganelles, determines the severity of clinical symptoms. In this study, we investigated the relationship between the main biomarker for peroxisomal diseases (VLCFA), and the severity of the disease measured as survival time. The five parameters were analysed in four patient’s groups. Our results have shown a high correlation between C26:0 and survival. Literature data from the description of the first patient shows that children with severe, classical form of ZS die within the first months to 6 m, the furthest to the first year of life [9]. Recently, there are more and more descriptions of patients with milder forms, who survive longer, even to adulthood [18].

Of course, the survival time is not an objective value and depends on many factors. Some of them are not even directly related to the disease. PDs belong to the group of rare diseases whose symptomatology includes a wide range of clinical symptoms caused by damage to many organs. Numerous morphological defects increase the patients’ susceptibility to childhood or infectious diseases. Clinical and parental care must be very extensive. Its quality cannot be overestimated and obviously has a large impact on the course of the disease and the length of the survival.

Since the high accumulation of VLCFA in body fluids and brains of patients with PBD and X-ALD has been found, they have been considered to be an important toxic factor in the pathogenesis of these diseases [19]. However, we do not know to what extent exactly, the excess of VLCFA affects the neurophysiological structure and processes of the nervous tissue. A simple, extremely long carbon chain with a one functional group, determines the high hydrophobicity, and low polarity of a VLCFA molecule, which implies its specific physicochemical properties, and consequently the physiological properties in the cell structure, other than long-chain fatty acids.

Studies have shown that VLCFA (mainly as C26:0) incorporated into the phospholipid cell membrane destabilises its structure and biochemical processes [20]. Addition of C26:0 to the adrenocortical cell culture medium leads to membrane microviscosity growth and decreases response to ACTH stimulation [21]. These results confirm the previously observed increase in membrane microviscosity in erythrocyte X-ALD patients [22]. Toxicity C26:0 has been demonstrated in several experiments. In vitro studies have demonstrated strong cytotoxic VLCFA activity in oligodendrocytes, astrocytes and rat neurons. C26:0 has particularly strong toxic effect, mainly in oligodendrocytes, inducing apoptosis [23]. In mice, injection of C24:0-lysophosphatidylcholine into the brain, resulted in broad activation of microglia and apoptosis [24]. Moreover, C26:0 exposure caused disturbance of intracellular Ca homeostasis, as well as depolarisation of the mitochondria in situ [23, 25].

In other studies, it was concluded that oxidative stress and mitochondrial disorders caused by VLCFA may be the result of axonal degeneration in the spinal cord [26, 27]. Increased production of ROS after exposure to VLCFA has been demonstrated in human fibroblasts and neuronal mouse cells. In the context of finding elevated lipids peroxidation products in X-ALD patients, the aforementioned conclusion indicates that oxidative stress can be involved in PD pathogenesis [25, 28, 29]. The disorder of peroxisomal lipid oxidation and the increase in ROS production destroy the mitochondrial functions in the mouse model ZS [30].

In turn, increased VLCFA incorporation in phospholipids of the internal mitochondrial membrane may interfere with OXPHOS complexes causing electron leakage and increased production of ROS, which will impair the energy status of the cell [31, 32]. The above data indicates that the disorder of peroxisomal lipid homeostasis can cause dysregulation of cell physiology, and may be multidirectional, in the cell membrane, redox status, metabolism, energy status, as well as it leads to neuropathological changes.

The results presented above confirm our previous reports in which we demonstrated a high correlation between the C26:0 level and the survival time (*r* = 0.822) for the PBD on a smaller group of patients (n = 9) [8]. Earlier, while analyzing categories of patients with PD, Moser et al. noticed relationship between VLCFA levels and severity of phenotype in PD [33]. Whereas, Gootjes et al. showed a dependence between the C26:0 β-oxidation measurement in cultured skin fibroblasts and the time of survival for ZSD. It has been suggested that besides DHAPAT, C26:0 level may be a good predictor for the severity of the disease [34]. Unlike our study, in which we proved that survival probability depends on of the C26: 0 level, Gootjes results’ based on demonstrating a statistically significant difference between two groups of children (deceased <1 year and alive> 5 years). In turn, Klouwer et al. examining sensivity of C26: 0-lysophosphatidylcholine (C26: 0-lysoPC) in the diagnosis of ZSD patients showed a medium negative correlation between C26:0-lysoPC levels and the age of the patient at the time of the analysis (r = -0.4258) [35]. Similarly, it has been previously reported that another peroxisomal diagnostics biomarker, i.e. plasmalogen, which is the major component of cell membranes, correlates with the disease severity of RCDP patients [36].

Although Berendse et al. notes that in the group of patients with mild, long-term survival ZSD, peroxisomal metabolites are varied, and therefore not correlated with the severity of the form. However, it mainly concerns DHCA, THCA and pipecolic acid. Only in 2/19 of patients, a reduction or normalisation of VLCFA levels was observed [10]. Our experience is different. Long-time monitoring of VLCFA levels in 4 patients (2 with ZS, DBP, and 1 with mild form ZSD) did not show a decrease in biomarker concentration. In a patient with mild form ZSD, over the course of 3 years, a stable, slightly increasing trend of VLCFA level was registered [11]. This data suggests that VLCFA is a stable diagnostic biomarker which can be used as a prognostic parameter.

The above works [34, 35] are based only on the patient’s ZSD. Our research group covers a wider spectrum of PDs ZSD, DBP and unspecified PD. In this study we investigated by comparative statistical analysis the correlation of 5 parameters of VLCFA levels and type of the syndrome with diseases severity defined as the survival. The results pointed to the fact that the probability for survival depends on C26:0 level and it is closely correlated with it (r = -0. 762). Indirectly these results confirm the toxic effect of VLCFA showing that increased concentration C26:0 is associated with a more severe phenotype of the disease and results in a shorter survival period.

On the other hand, VLCFA can be applied as a parameter for monitoring the patient’s therapeutic course. The sequential surveillance of VLCFA levels after HSCT, is important tool for the assessment of engraftment, graft failure or rejection, and could be useful in the treatment effectiveness [37]. At the current stage of knowledge, we do not know how to treat these diseases. Various treatment attempts are being undertaken. Detection of VLCFA levels is not always used to monitor of therapeutic effects, but its analysis provides interesting information [8, 37–39].

## Conclusion

VLCFA levels correlate with the severity of the clinical course of ZS, DBP and mild ZS. The best predictive value for projecting a severity of the disease is the concentration of C26:0. Survival probability is correlated with C26:0 level. This, in turn, indirectly confirms the toxic effect of this metabolite on the organism. Research involving the implementation of medicines, as well as of other medical techniques for therapeutic purposes for this group of diseases, should include the VLCFA level monitoring.
